# Circulating 25-hydroxycholecalciferol and calcium levels, and alkaline phosphatase activity among people living with and without human immunodeficiency virus and injecting drugs in kenya

**DOI:** 10.1186/s12879-024-09610-8

**Published:** 2024-07-17

**Authors:** Abel O. Onyango, Nathan Shaviya, Valentine Budambula, George O. Orinda, Omu Anzala, Ahmed A. Aabid, Tom Were

**Affiliations:** 1 Department of Biochemistry, Microbiology, and Biotechnology, Kenyatta University, P. O. Box 43844-00100, Nairobi, Kenya; 2 Department of Medical Laboratory Sciences, Masinde Muliro University of Science and Technology, P. O. Box 190-50100, Kakamega, Kenya; 3 Department of Environment and Health, Technical University of Mombasa, GPO Mombasa, P. O. Box 90420-80100, Mombasa, Kenya; 4 Kenya AIDS Vaccine Initiative - Institute of Clinical Research, University of Nairobi, P. O. Box 30197-00100, Nairobi, Kenya; 5 Bomu Hospital, P.O. Box 95683-80106, Mombasa, Kenya; 6 Department of Medical Microbiology and Parasitology, Masinde Muliro University of Science and Technology, P. O. Box 190-50100, Kakamega, Kenya

**Keywords:** 25-hydroxycholecalciferol, Calcium, Alkaline phosphatase, People-who-inject-drugs, People-living-with-HIV

## Abstract

**Background:**

People who inject drugs (PWID) and living with the human immunodeficiency virus (PLHIV) are at higher risk of suffering marked derangements in micronutrient levels, leading to poor disease and treatment outcomes. Consequently, this can be monitored by measuring key biomarkers, such as total circulating (serum) 25-hydroxycholecalciferol (25(OH)D_3_), calcium, and alkaline phosphatase (ALP) for timely intervention. Therefore, circulating levels of 25(OH)D_3_ and calcium, and ALP activity were determined in PWID and are highly active anti-retroviral treatment (HAART)-experienced or -naive, along with those without HIV infection.

**Methods:**

This cross-sectional study compared serum concentrations of 25(OH)D_3_, calcium, and ALP in Kenyan PLHIV and were HAART-naive (*n* = 30) or -experienced (*n* = 61), PWID and without HIV (*n* = 132).

**Results:**

Circulating 25(OH)D_3_ levels were significantly different amongst the study groups (*P* < 0.001), and were significantly lower in the HAART-experienced (median, 17.3; IQR, 18.3 ng/ml; *P* < 0.001) and -naive participants (median, 21.7; IQR, 12.8 ng/ml; *P* = 0.015) relative to uninfected (median, 25.6; IQR, 6.8 ng/ml) PWID. In addition, the proportions of vitamin D deficiency (55.7%, 40.0%, and 17.4%) and insufficiency (31.1%, 53.3%, and 63.6%) compared to sufficiency (13.1%, 6.7%, and 18.9%; *P* < 0.001) were greater amongst HAART-experienced, -naive, and uninfected study groups, respectively. Likewise, serum total calcium concentrations were lower in the HAART-experienced relative to HIV-negative (*P* = 0.019) individuals. Serum ALP activity was also lower in the HAART-experienced in contrast to HIV-negative PWID (*P* = 0.048). Regression analysis indicated that predictors of circulating 25(OH)D_3_ were: age (β = 0.287; R^2^ = 8.0%; *P* = 0.017) and serum ALP (β = 0.283; R^2^ = 6.4%; *P* = 0.033) in the HAART-experienced PWID, and serum ALP (β = 0.386; R^2^ = 14.5%; *P* < 0.001) in the HIV-negative PWID.

**Conclusion:**

This study suggests that HIV-1 infection and HAART, including injection substance use, decrease circulating 25(OH)D_3_, calcium and ALP activity. In addition, age and ALP activity are associated with low circulating vitamin D levels in HAART-experienced PWID. The results highlight the importance of incorporating vitamin D and calcium supplementation in treatment and rehabilitation protocols for PLHIV.

## Background

People living with HIV (PLHIV) and those injecting illicit drugs suffer marked micronutrient and macronutrient deficiencies [1–3]. Vitamin D is one of the most important micronutrients altered in PLHIV and those injecting drugs [4–6]. Adequate circulating vitamin D levels in the body are critical in modulating clinical outcomes of HIV infection [7, 8]. Vitamin D is a key regulator of bone homeostasis [9, 10]. In addition, it is involved in regulating immune responses such as the activation of cell-mediated immunity, suppression of leucocyte proliferation, monocyte activation, and cytokine production [11]. Vitamin D deficiency has been reported in PLHIV [12, 13], and this is associated with low intake, as well as the use of efavirenz, nevirapine, tenofovir and ritonavir containing antiretroviral regimens [4, 14]. Nonetheless, supplementation restores vitamin D status, calcium, and alkaline phosphatase (ALP) activity [15]. However, it is not clear how concurrent HIV infection, highly active antiretroviral therapy (HAART) and injecting drug use influence vitamin D status.

Calcium is important in bone mineralisation, but previous studies reported decreased serum calcium levels in PLHIV [15]. HIV infection alters bone metabolism through inflammatory responses [16]. For instance, pro-inflammatory cytokines such as tumour necrosis (TNF)-α function by inhibiting osteoblasts and activating osteoclasts, hence elevating circulating levels of calcium [17]. Although elevated serum calcium independent of low vitamin D levels has been reported in heroin addicts [18], the effect of substance use on circulating calcium levels is not clear.

ALP is an enzyme produced in the liver and osteoblasts that hydrolyses phosphate esters releasing inorganic phosphate, and serum ALP activity is elevated in PLHIV initiated on HAART presenting with severe hepatotoxicity [19]. Likewise, elevated serum ALP activity predicts the degree of hepatic inflammation in chronic hepatitis B infection and marijuana-induced hepatotoxicity, as well as hepatobiliary and bone diseases [20–23]. In addition, previous studies on PLHIV showed that elevated serum ALP activity was associated with immunodeficiency (CD4 count < 200 cells/µl), laboratory markers of bone turnover, and non-nucleoside reverse-transcriptase inhibitors (NNRTI; nevirapine and efavirenz) use [24, 25]. Furthermore, use of nucleoside reverse transcriptase inhibitors (NRTIs) such as tenofovir, co-morbidities and demographic factors has also been associated with alterations in serum ALP activity in PLHIV [24]. Nevertheless, no clear mechanisms have been put forth to explain serum ALP elevation in HAART-naive and -experienced PWID living with HIV.

Serum 25-hydroxycholecalciferol (also known as calcifediol or calcidiol and abbreviated as (25(OH)D_3_) concentrations are a summation of vitamin D intake and sunlight exposure synthesised vitamin D, and as such is used as a biomarker of the overall vitamin D status because of a longer half-life of 2–3 weeks compared to 4–6 h for 1,25-dihydroxycholecalciferol or calcitriol (1,25-(OH)_2_D_3_) [26]. Vitamin D from sunlight exposure, diet, and supplements is hydroxylated in the liver to 25(OH)D_3_ and in the kidneys to generate the active form 1,25-(OH)_2_D_3_ [27], which promotes calcium and phosphate conservation [28]. Altogether, it appears that the homeostatic balance of these bone mineralisation markers is markedly altered in PWID and living with HIV. However, there are no reports from Kenya on the interrelationships of serum 25(OH)D_3_, calcium, and ALP in PWID and are living with HIV. Therefore, it is possible that the increasing population of PWID in Kenya with a high burden of HIV infection suffer marked pathophysiologic derangements which can influence strategies of management. Therefore, this study examined the interrelationship of serum 25(OH)D_3_ with calcium and ALP in HAART-experienced or -naive, and HIV-negative PWID.

## Methods

***Selection and description of participants.*** This cross-sectional study was conducted as part of a larger study investigating the demographic and laboratory factors associated with HIV infection amongst PWID in Mombasa, a coastal city in Kenya. A detailed description of the study site and the population is presented in our previous publications [29–32]. A total sample size of 223 serum specimens from PWID was estimated [33] based on a margin of error of 5%, confidence interval of 95%, response distribution of 82.3%, and a population of 49,167 PWID in Kenya [34]. The sample size was then stratified according to HIV prevalence of 41% in PWID [35], and HAART cover of 0.67% in PWID living with HIV [36]. Thus, the following three groups of PWID were analysed: (1) HIV-negative (*n* = 132); (2) HAART-naive (*n* = 30); and (3) HAART-experienced (*n* = 61). The HAART-naive were individuals newly diagnosed with HIV infection. The HAART-experienced were individuals on HAART, and HIV-negative comprised PWID testing negative for HIV infection. Demographic information, substance use profile, body mass index (BMI), CD4 + T cell counts, including HIV screening and viral load determinations, and sample collection procedures were previously described [31, 37].

***Serum 25-hydroxycholecalciferol***,*** calcium***,*** and alkaline phosphatase activity***. About 10 ml of blood samples were collected by venepuncture into plain vacutainer tubes containing a clot activator and used for serum preparation. The serum samples were aliquoted, and stored frozen at -70^o^ C until used for batched analyte measurements. Automated clinical chemistry and immunoassay analyser (ROCHE COBAS^®^ e601 and e501, Lausanne, Switzerland) respectively were used for batched measurements of 25(OH)D_3_, and total calcium, while DIRUI CS-4000 (Dirui Industrial Company ltd., Changchui, China) auto-chemistry analyser was used for determining ALP activity. Serum 25(OH)D_3_ was used for measuring vitamin D status because of a longer half-life than the 1,25-(OH)_2_D_3_ [26]. In the Arsenazo method, total serum calcium is determined at an acidic pH which frees complexed and albumin-bound calcium for specific binding of calcium ions to arsenazo III (2,2’-[1,8-dihydroxy-3,6-disulphonaphthylene-2,7-bisazo]bisbenzenear-sonic acid). The intensity of the purple-coloured reaction product is proportional to the concentration of total calcium present in the sample and was quantified by colorimetry. In addition, calcium estimation was based on clinical practice standards [38], as the method measures both bound and free calcium. The ROCHE COBAS^®^ reagents were supplied by Roche diagnostics through a local subsidiary (Sciencescope ltd., Nairobi, Kenya). Quality control assays for 25(OH)D_3_ and calcium were performed prior to analysing the samples. All analyses were carried out in accordance with the principles of good clinical laboratory practices.

***Statistical analysis.*** Statistical data analysis was conducted using IBM SPSS Statistics for Windows, Version 25.0. Armonk, NY: IBM Corp. Age, weight, height, BMI, CD4 + T cell counts, HIV-1 RNA copies, 25(OH)D_3_, calcium, and ALP were compared across the study groups using Kruskal Wallis U tests followed by Dunn’s post-hoc corrections. Distributions of gender, BMI, immune, HIV-1 viraemia, 25(OH)D_3_, calcium, and ALP status were compared amongst the study groups using the Pearson’s chi-square tests. To determine the prevalence of micronutrient deficiency, the serum concentrations of 25(OH)D_3_ were categorised as sufficient (≥ 30 ng/ml), insufficient (21–29 ng/ml) and deficient (< 20 ng/ml) [39]; total calcium was stratified into hypocalcaemia (< 2.2 mmol/L) and hypercalcaemia (> 2.6 mmol/L), whereas ALP was categorised into low ALP activity (< 53.0 IU/L) as previously established for Kenyan adults [40]. Linear hierarchical regression modelling was performed to determine the predictors of circulating 25(OH)D_3_ concentrations. First, the variables were log-transformed towards normality before regression modelling each study group of PWID. In all the study groups, serum concentrations of 25(OH)D_3_ were entered as the dependent variable. Calcium plus age, CD4 + T cells, HIV-1 RNA copies, and BMI or age, CD4 + T cells, and BMI were entered in the models as the predictor variables for the PWID and were HAART-experienced, -naive, and were HIV-negative, respectively. All tests were two-tailed with statistical significance set at *P* < 0.05.

## Results

***Demographic***,*** drug use***,*** and clinical profiles of the study participants***. The demographic and drug use profiles of the study participants are presented in Table 1. The median age distribution was not significantly different (*P* = 0.441) across the study groups. Gender distribution differed significantly across the study groups (*P* < 0.001), and the HAART-experienced group had more females (*n* = 38; 62.3%) relative to HAART-naive (*n* = 13; 43.3%) and HIV-negative (*n* = 12; 9.1%) PWID. Body height (m) was significantly different in between the study groups (*P* < 0.01). CD4 + T cell counts (/µl) were significantly different across groups (*P* < 0.001) with HAART-experienced (*P* < 0.001) and HAART-naive (*P* < 0.001) participants presenting with lower counts compared to HIV-negative individuals. In addition, proportions of immune suppression (CD4 + T cell counts < 500.0/µl) were (*n* = 41; 67.2%) in the HAART-experienced, -naive (*n* = 15; 50.0%), and HIV-negative (*n* = 26; 19.7%; *P* < 0.001) PWID. Heroin was the most frequently injected substance in all study groups but the proportion of users varied among the study groups [HAART-experienced (*n* = 42; 68.9%); HAART-naive (*n* = 22; 73.3%) compared to the HIV-negative group (*n* = 120; 90.9%); *P* < 0.001]. Injection cocaine use was reported in less than 30.0% of the HIV-infected groups [HAART-experienced (*n* = 18; 29.5%); HAART-naive (*n* = 7; 23.3%) compared to the HIV-negative group (*n* = 9; 6.8%); *P* < 0.001]. Concomitant injection of cocaine and heroin was reported in less than 3.5% of the study participants [HAART-experienced (*n* = 1; 1.6%); -naive (*n* = 1; 3.3%); and HIV-negative (*n* = 3; 2.3%) individuals]. Frequency of drug injection (> twice a day) was higher in HAART-experienced (*n* = 50; 82.0%) and -naive (*n* = 19; 63.3%) individuals compared to the HIV-negative (*n* = 72; 54.5%; *P* = 0.001] PWID. Finally, the duration of injection (> 1 year) was also higher in the HAART-experienced (*n* = 55; 90.2%) and -naive (*n* = 27; 90.0%) patients relative to the HIV-negative (*n* = 81; 61.4%; *P* < 0.001) individuals.

**Table 1:** Demographic and clinical profiles of the study participants

Characteristic	HIV[-]/HAART[-], *n* = 132	HIV[+]/HAART[-], *n* = 30	HIV[+]/HAART[+], *n* = 61	*P*
Age, yrs.	32.3 (9.9)	30.7 (7.6)	30.3 (8.7)	0.441
Female, n (%)	12 (9.1)	13 (43.3)	38 (62.3)	**< 0.001**
Weight, kg	54.5 (8.8)	54.0 (8.8)	53.0 (7.0)	0.051
Height, m	1.7 (0.1)	1.7 (0.1)	1.7 (0.1)^a^	**0.005**
BMI, kg/m^2^	18.7 (2.8)	18.7 (2.6)	18.8 (2.4)	0.984
BMI < 18.5 kg/m^2^	73 (44.7)	16 (36.7)	34 (44.3)	0.975
CD4 + T cells/µl	937.0 (618.0)	495.0 (369.0)^c^	357.0 (317.5)^b^	**< 0.001**
<500 CD4 + T cells/µl	26 (19.7)	15 (50.0)	41 (67.2)	**< 0.001**
Log_10_ HIV-1 RNA copies/ml	…	3.5 (2.4)	2.9 (2.4)	0.451
≥1000 Log_10_ HIV-1 RNA copies/ml	…	16 (59.3)	29 (48.3)	0.345
Heroin	120 (90.9)	22 (73.3)	42 (68.9)	**< 0.001**
Cocaine	9 (6.8)	7 (23.3)	18 (29.5)	**< 0.001**
Cocaine and heroin	3 (2.3)	1 (3.3)	1 (1.6)	…
Frequency of injection > 2/day	72 (54.5)	19 (63.3)	50 (82.0)	**0.001**
Duration of injection > 1 year.	81 (61.4)	27 (90.0)	55 (90.2)	**< 0.001**

Data are presented as number (n) and proportion (%) of participants for categorical variables, and as medians (interquartile range) for age, CD4 T cell counts and log_10_ HIV RNA copies. HIV, human immunodeficiency virus. HAART, highly active antiretroviral treatment. Across group comparisons were performed using the Pearson’s chi-square for proportions, and Kruskal Wallis tests for age, weight, height, BMI, and CD4 + T cell counts. Post-hoc Dunn’s test for multiple comparisons was performed for height and CD4 + T cell counts. HIV RNA copies were compared between the HAART-experienced and -naive groups using the Mann-Whitney U test. ^a^*P* = 0.004, ^b^*P* < 0.001, and ^c^*P* < 0.001 vs. uninfected people-who-inject-drugs. Values in bold are significant *P-*values

***Serum 25-hydroxycholecalciferol***,*** total calcium and alkaline phosphatase activity.*** The circulating concentrations of 25(OH)D_3_, calcium and ALP are shown in Table 2. The serum 25(OH)D_3_ levels differed significantly amongst the study groups (*P* < 0.001). Post-hoc analysis indicated that serum 25(OH)D_3_ levels were significantly lower in the HAART-experienced (*P* < 0.001) and -naive (*P* = 0.015) PWID relative to the HIV-negative PWID. Consistent with lower 25(OH)D_3_ levels, the prevalence of vitamin D deficiency was higher in the PLHIV and HAART-experienced (*n* = 34; 55.7%) vs. the -naive (*n* = 12; 40.0%) and HIV-negative (*n* = 23; 17.4%) PWID. However, proportions of 25(OH)D_3_ insufficiency were lower in the HAART-experienced (*n* = 19; 31.1%) compared to -naive (*n* = 16; 53.3%) and HIV-negative (*n* = 84; 63.6%) PWID. Accordingly, the overall prevalence of 25(OH)D_3_ sufficiency was low in the study groups: HAART-experienced (*n* = 8; 13.1%), HAART-naive (*n* = 2; 6.7%), and HIV-negative (*n* = 25; 18.9%; *P* < 0.001) PWID. Serum total calcium levels were significantly (*P* = 0.023) different across the study groups. Post-hoc analysis showed that serum total calcium concentrations were lower in HAART-experienced PWID in comparison to HIV-negative (*P* = 0.019) participants. Consistent with low calcium concentrations, a higher prevalence of hypocalcaemia was present in HAART-experienced (*n* = 22; 36.1%) vs. HAART-naive (*n* = 9; 30.0%) and HIV-negative (*n* = 33; 25.0% PWID. As a result, low prevalence of hypercalcaemia was present in all the study groups [HAART-experienced (*n* = 4; 6.6%); -naive (*n* = 1; 3.3%; and HIV-negative (*n* = 7; 5.3%; *P* = 0.532)]. Likewise, serum activity of ALP was significantly different amongst the study groups (*P* = 0.049). Post-hoc analyses showed that serum ALP activity was lower in the HAART-experienced PWID relative to the HIV-negative PWID (*P* = 0.048). In addition, no high ALP activity was noted in the study groups [HAART-experienced (*n* = 0; 0.0%); HAART-naive (*n* = 0; 0.0%); and HIV-negative (*n* = 0; 0.0%) PWID].

**Table 2:** Circulating levels of vitamin D, calcium, and alkaline phosphatase activity

Analyte	HIV(-)/HAART(-), *n* = 132	HIV(+)/HAART(-), *n* = 30	HIV(+)/HAART(+), *n* = 61	*P*
25-hydroxycholecalciferol, ng/ml	25.6 (6.8)	21.7 (12.8)^b^	17.3 (18.3)^a^	**< 0.001**
Deficiency	23 (17.4)	12 (40.0)	34 (55.7)	**< 0.001**
Insufficiency	84 (63.6)	16 (53.3)	19 (31.1)	
Sufficiency	25 (18.9)	2 (6.7)	8 (13.1)	
Calcium, mmol	2.3 (0.2)	2.3 (0.2)	2.2 (0.2)^c^	**0.023**
Hypocalcaemia	33 (25.0)	9 (30.0)	22 (36.1)	0.532
Hypercalcaemia	7 (5.3)	1 (3.3)	4 (6.6)	
ALP, U/L	61.0 (33.3)	55.0 (26.0)	53.5 (27.5)^d^	**0.049**
>153 U/L	0 (0.0)	0 (0.0)	0 (0.0)	….

Data are presented as medians (interquartile range) for continuous variables and numbers (proportions) of participants for categorical variables. HIV, human immunodeficiency virus. HAART, highly active antiretroviral treatment. ALP, alkaline phosphatase. Deficiency, 25-hydroxycholecalciferol (25(OH)D_3_) < 20 ng/ml; insufficiency, 25(OH)D_3_ 20–29 ng/ml; sufficiency, 25(OH)D_3_ ≥ 30 ng/ml [39]. Hypocalcaemia (calcium < 2.2 mmol/L) and hypercalcaemia (calcium > 2.6 mmol/L). High ALP activity (ALP > 153.0 U/L for males of all ages, and ALP > 130.0 and > 170 U/L for females < 45 years and ≥ 45 years old) [40]. Data analysis was conducted using the Pearson’s chi-square for proportions and Kruskal Wallis tests for 25(OH)D_3_, calcium, and ALP levels across the groups. Post-hoc Dunn’s test for multiple comparisons: ^a^*P* < 0.001, ^b^*P* = 0.015, ^c^*P* = 0.0194, and ^d^*P* = 0.048 vs. uninfected people-who-inject-drugs. Values in bold are significant *P*-values

***Predictors of circulating 25-hydroxycholecalciferol concentrations.*** Hierarchical linear regression modelling for predictors of circulating 25(OH)D_3_ concentrations amongst the HAART-experienced individuals was significant (F (6, 54) = 3.661, *P* = 0.004) with the entire set of variables (total calcium plus age, ALP, CD4 + T cells, BMI and HIV-1 RNA copies) accounting for 28.9% of the variance in circulating 25(OH)D_3_ levels (*R* = 0.538, R^2^ = 0.289). In addition, age (β = 0.287, *P* = 0.017), and ALP (β = 0.283, *P* = 0.033) were associated with the 25(OH)D_3_ concentrations. Squared semi-partial correlations revealed that the unique 25(OH)D_3_ concentrations accounted for by age, and ALP was 8.0%, and 6.4%, respectively. The modelling for the predictors of 25(OH)D_3_ levels in the HAART-naive individuals was, however, not significant (F (6, 23) = 1.170, *P* = 0.356; (*R* = 0.484, R^2^ = 0.234)). Furthermore, hierarchical regression modelling for 25(OH)D_3_ concentrations in the HIV-negative individuals was significant (F (5, 126) = 5.026, *P* < 0.001) with the entire set of variables (calcium plus age, ALP, CD4 + T cells, and BMI) accounting for 16.6% of the difference in the circulating 25(OH)D_3_ concentrations (*R* = 0.408, R^2^ = 0.166). Besides, ALP activity (β = 0.386, *P* < 0.001) was significantly associated with the 25(OH)D_3_ concentrations. Squared semi-partial correlations indicated that the unique quantity of variance in 25(OH)D_3_ concentrations accounted for by ALP was 14.5%.

## Discussion

The lower levels, including higher proportions of deficiency and insufficiency of serum 25(OH)D_3_ in the HAART-naive and -experienced PWID, suggest HIV infection and substance use exacerbation in vitamin D deficiency. These findings are consistent with previous studies showing lower concentrations of vitamin D and high proportions of vitamin D deficiency in PWID living with or without HIV [4–6]. The underlying mechanisms for the low levels of vitamin D status in PWID include HIV infection- and substance-induced chronic inflammation and immunological hyperactivity. This is further emphasised by results indicating that low levels of vitamin D are associated with seropositivity for hepatitis C virus and HIV-infections, both of which are common chronic inflammatory-associated co-morbidities in PWID [4]. The role of inflammation in suppressing the vitamin D status, is also possibly related to a shift in the oxidative and anti-oxidative balance [41] and over-secretion of inflammatory mediators such as TNF-α interfering with production of 25(OH)D_3_ resulting in vitamin D deficiency [42]. Additionally, hepatic injury and altered metabolism can lead to vitamin D deficiency given that antiretroviral drugs such as lopinavir/ritonavir, tenofovir disoproxil fumarate and efavirenz are associated with low vitamin D levels in PLHIV [43, 44]. Consistent with these observations, protease inhibitors, NRTIs and NNRTIs promote hydroxylation of vitamin D and its metabolites to biologically inactive compounds, leading to vitamin D deficiency [45, 46]. Moreover, opioids, antiretroviral drugs, and 25-(OH)D are also metabolised via the cytochrome P450 system [47, 48], resulting in interactions that possibly alter the availability of 1,25-(OH)_2_D_3_.

In the present study, age, calcium, ALP, CD4 + T cell count and viral load were the key predictors of serum 25-(OH)D concentrations in the study groups. These findings, in part, mirror previous studies in the USA, India, Australia, and Kenya indicating that age, low dietary intake of calcium, CD4 + T cells, viral load, opioid dependence and markers of liver injury, such as alanine aminotransferase, ALP and hypoalbuminaemia in PLHIV HAART-experienced non-injecting drug users and PWID [4, 6, 14, 49, 50]. Furthermore, low vitamin D concentrations are common in people of black ethnicity, such as African and black American PLHIV [6, 51]. Therefore, a complex interplay of multiple risk factors influences the development of vitamin D deficiency and insufficiency in PWID.

Hypocalcaemia is common in HAART-naive and -experienced PLHIV [52, 53]. Consistent with previous findings, our study found lower median serum calcium levels in PLHIV HAART-experienced PWID. Previous studies indicated that PWID are largely at a high risk of under-nutrition [54]. This is possibly due to low dietary intake and limited finances, since available resources are primarily used to sustain the drug habit [2, 55]. Consequently, this contributes to the low serum calcium concentrations observed in PWID. Additionally, low calcium levels have been associated with low vitamin D concentrations, since vitamin D enhances absorption of dietary calcium [56]. Likewise, infection with HIV often leads to hypoparathyroidism [56], which is associated with hypocalcaemia. Since a majority of the study participants were using heroin, it is possible to conclude that they would present with low serum calcium levels and subsequently higher proportions of hypocalcaemia.

Although ALP appears to have no clinical utility in PWID and PLHIV, previous studies showed higher serum ALP levels in opioid-dependent individuals [57], and predicted the degree of hepatotoxicity in patients on HAART in Cameroon at one month and six-month follow-ups [19]. However, the current study found that serum ALP activity was reduced in the HAART-experienced PWID, which may be attributed to the polysubstance use in this population. Nonetheless, few studies have examined serum ALP and other hepatic enzyme activities in the context of HAART-experienced HIV infected PWID. It is important to note that our previous studies indicated elevated serum aminotransferases in HAART-experienced people-who-inject-heroin [31], and historical studies over three decades ago found that at least 18% of cocaine users had elevated serum ALP activity [58]. As such, we are proposing that polysubstance use elicits varied hepato-pathophysiological effects in PWID, warranting further investigations.

Altogether, concomitant reduction in vitamin D, calcium and ALP amongst HAART-experienced PWID suggests that substance use, HIV-infection and ARVs directly and/or indirectly alter the delicate balance of vitamin D, calcium and ALP homeostasis in these patients. It appears that mechanistically, the low levels of vitamin D drive the suppression of intestinal, renal, and bone calcium mobilisation [59]. The implications of the dysfunction in these feedback loops include revision of the clinical protocols regarding renal, liver and bone mineral function in PLHIV and injecting drugs. One of the strengths of the present study was the concurrent approach to the measurement and linking of vitamin D status with calcium and phosphate concentrations in HAART-experienced PWID. The limitations of this study are, absence of urine analyses for metabolites of vitamin D and substances used, including the effect of genetic variability in the vitamin D receptors as this would have enabled linking with vitamin D status. Another limitation of this study is the fact that this was a cross-sectional design. A prospective approach would be useful in understanding the dynamics of vitamin D status including, adherence to HAART and injecting drug cessation. However, the current comparisons of HAART-experienced with HAART-naive and HIV-negative PWID provide valuable insights into the complex pathophysiologic mechanisms of HIV infection in PWID. The laboratory analysis for vitamin D status was based on batched automated measurement of 25(OH)D_3_ concentrations, and the use of local reference ranges which were consistent in this population. Even though our design was cross-sectional, and possibly limited by confounders from self-reported substance use duration, age, an important predictor of substance use in coastal Kenya [60], was used as a proxy for duration of substance use in the regression analyses. Besides, our study population was drawn from a southern latitude area amongst the native population of the coastal city of Mombasa, Kenya, and hence the findings are not generalisable to northern latitude populations.

## Conclusion

The present study therefore suggests that HIV-1 infection, HAART and injection drug use concomitantly reduce vitamin D levels, calcium and ALP in PWID. Additionally, age and serum ALP activity are associated with low circulating vitamin D status in PLHIV injecting drugs and initiated on HAART. The findings of this study highlight the need for policy review on monitoring, supplementation, and rehabilitation of PLHIV injecting drugs. Further research is recommended to evaluate the effects of newer HAART regimens on serum 25(OH)D_3_ in similar cohorts.

## Data Availability

The datasets used and/or analysed during the current study are available from the corresponding author on reasonable request.

## Abbreviations

ALP: Alkaline phosphatase
BMI: Body mass index
CD4: Clusters of differentiation 4
HAART: Highly active antiretroviral therapy
HIV: Human immunodeficiency virus
IBM: International business machines
IQR: Interquartile range
NNRTI: Non-nucleoside reverse-transcriptase inhibitor
PWID: People-who-inject-drugs
PLHIV: People living with HIV
RNA: Ribonucleic acid
SPSS: Statistical Package for the Social Sciences
